# Ether Oxidation by an Evolved Fungal Heme-Peroxygenase: Insights into Substrate Recognition and Reactivity

**DOI:** 10.3390/jof7080608

**Published:** 2021-07-28

**Authors:** Raul Mireles, Joaquin Ramirez-Ramirez, Miguel Alcalde, Marcela Ayala

**Affiliations:** 1 Departamento de Ingeniería Celular y Biocatálisis, Instituto de Biotecnología Universidad Nacional Autónoma de México, Cuernavaca 62210, Morelos, Mexico; raulmireleslopez@gmail.com (R.M.); joaquin.ramirez@ibt.unam.mx (J.R.-R.); 2 Department of Biocatalysis, Institute of Catalysis and Petrochemistry, CSIC, 28049 Madrid, Spain; malcalde@icp.csic.es

**Keywords:** fungal peroxygenase, biodegradation, suicide inactivation, ether oxidation, xenobiotic transformation

## Abstract

Ethers can be found in the environment as structural, active or even pollutant molecules, although their degradation is not efficient under environmental conditions. Fungal unspecific heme-peroxygenases (UPO were reported to degrade low-molecular-weight ethers through an H_2_O_2_-dependent oxidative cleavage mechanism. Here, we report the oxidation of a series of structurally related aromatic ethers, catalyzed by a laboratory-evolved UPO (PaDa-I) aimed at elucidating the factors influencing this unusual biochemical reaction. Although some of the studied ethers were substrates of the enzyme, they were not efficiently transformed and, as a consequence, secondary reactions (such as the dismutation of H_2_O_2_ through catalase-like activity and suicide enzyme inactivation) became significant, affecting the oxidation efficiency. The set of reactions that compete during UPO-catalyzed ether oxidation were identified and quantified, in order to find favorable conditions that promote ether oxidation over the secondary reactions.

## 1. Introduction

The ether functional group is commonly found in nature as metabolites, structural polymers, oil-derivatives or bioplastics, among others. They also can be found as active molecules in many man-made products, such as agrochemicals, cosmetics, detergents or drugs [1,2]. Several of these molecules have been reported to be present in water and soil as a consequence of inappropriate handling during their life cycle [3,4]. Some of them can be identified as pollutants due to their characteristic capability to remain chemically intact and still active in environmental conditions [5] (EPA, 2009). Their recalcitrant character is mainly due to the high stability of the ether bond. For instance, the energy needed for C-O bond dissociation in ethyl propyl ether (84.8–85.3 kcal/mol) is similar to that required for a C–C cleavage in the same molecule (85.3 kcal/mol) [6].

Although ethers are widely found in the environment, their degradation is not a very common reaction in nature. In recent years, the description of a fungal heme-thiolate peroxidase (termed unspecific peroxygenase, UPO: E.C. 1.11.2.1) has uncovered a whole family of enzymes that is known for catalyzing the mono(per)oxygenation of over 350 organic molecules, with particular preference for hydrophobic, aromatic substrates [7,8]. It is noteworthy that these enzymes appear to be present solely in fungi. Although the biological role of these enzymes has not been elucidated, several works highlight their ability to catalyze the oxidation of hundreds of organic compounds. Interestingly, UPO catalyzes the degradation of ether molecules via the oxidative cleavage of the ether bond. In this H_2_O_2_-dependent mechanism, an oxygen atom is inserted to the carbon adjacent to the oxygen atom in the ether functional group; the product (a hemiacetal) is chemically unstable and leads to spontaneous ether bond breakage to generate an alcohol and an aldehyde [9]. In contrast, cytochrome P450 is able to catalyze aromatic O-demethylation [10,11].

Evidence regarding hydrophobic aliphatic (both linear and cyclic) ether oxidation has been reported previously [9,12,13]. In this work, a series of aromatic ethers was selected in order to analyze if substituents with different polarity affect ether oxidation catalyzed by a laboratory-evolved version (PaDa-I mutant) of UPO from *Agrocybe aegerita*. We found that aromatic ethers are not efficiently transformed by PaDa-I; thus, the magnitude of secondary, unproductive reactions occurring simultaneously during aromatic ether oxidation was quantified, in order to adjust reaction conditions so that ether oxidation was favored.

## 2. Experimental Procedure

### 2.1. Chemicals and Reagents

Benzyl ethers, 5-nitro-1,3-benzodioxole (NBD), 2,2′-azino-bis(3-ethylbenzothiazoline-6-sulfonic acid (ABTS), and H_2_O_2_ were purchased from Sigma. Acetonitrile for HPLC analysis and salts for buffer solutions were obtained from JT Baker. PaDa-I was heterologously expressed and purified [14] to >95% of electrophoretic homogeneity and a Reinheitszahl value (R_z_ = A_407_ nm/A_280_ nm) of 2.1. Culture media reagents were purchased from Difco.

### 2.2. Enzyme Reactions

Ether oxidation was monitored by liquid chromatography, using a reverse phase Phenomenex^®^ Luna C18 column (150 mm × 3 mm, 5 µm particle size) in an Agilent 1220 Infinity HPLC (Santa Clara, CA, USA) coupled to a UV-Vis detector, set to 210 nm. Analysis was performed using the following acetonitrile-water (ACN/H_2_O) gradient in % *v/v*: ACN:H_2_O 10:90 for 5 min, a 9 min gradient to reach ACN:H_2_O 100:0, maintaining 4 min at 100:0. The retention times (t_R_) were 12.8 min for 2-(benzyloxy)ethanol, 17.4 min for allylbenzylether, 18.2 min for benzyloxyacetaldehyde and 18.5 min for dibenzylether. Mobile phase for benzyloxyacetate was a 10 min ACN/PA gradient set from 20:80 to 80:20. Column flow was 0.4 mL/min and the temperature was set to 30 °C.

**Figure 1:**
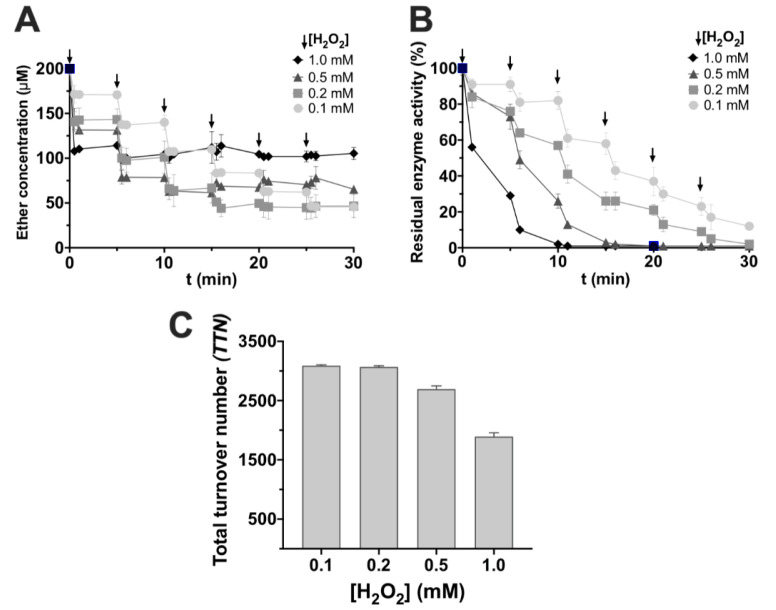
(**A**) 2-(benzyloxy)ethanol consumption. (**B**) Enzyme inactivation in the presence of 0.2 mM substrate and different additions of H_2_O_2_. Arrows indicate H_2_O_2_ additions to the reaction mixture. (**C**) Total turnover number (TTN) for the oxidation of 2-(benzyloxy)ethanol, at different H_2_O_2_ dosages (initial concentration of substrate, 0.2 mM).

**Figure 2:**
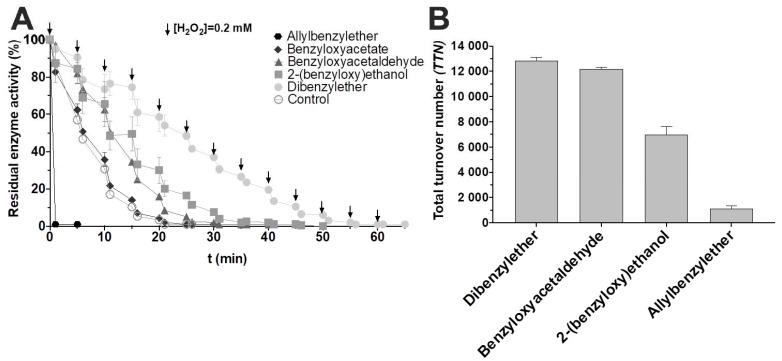
(**A**) Residual activity for PaDa-I in the presence of selected ethers. (**B**) TTN for selected ether compounds. These assays were performed at the maximum substrate solubility (0.7 mM) with 0.2 mM H_2_O_2_ additions.

The ether oxidation reaction mixture was typically composed of 0.2 (Figure 1) or 0.7 mM (Figure 2) substrate and 50 nM PaDa-I in a 50 mM potassium phosphate buffer solution pH 7.0 containing 20% *v*/*v* acetonitrile, in a total volume of 1 mL. Total turnover number (TTN) was calculated as the mol of substrate converted per mol of enzyme, until the enzyme is completely inactive. Reactions were initiated by adding 0.1–1.0 mM H_2_O_2_. H_2_O_2_ was added when the reaction (according to substrate consumption) halted. Aliquots (10–50 μL) were taken after every H_2_O_2_ addition in order to measure residual enzyme activity. Thus, enzyme inactivation was estimated as the residual peroxidase activity in a reaction mixture containing 0.4 mM ABTS in 100 mM sodium phosphate/citrate buffer pH 4.4 and 2.0 mM H_2_O_2_. Residual activity was assayed in a 1.0 mL quartz cuvette. ABTS oxidation was followed spectrophotometrically (ε_418_ = 36,000 M^−1^cm^−1^) [15] in an Agilent 8453 UV-Visible Spectroscopy System (Santa Clara, CA, USA). For determining the influence of substrate concentration on TTN, the concentration of 2-(benzyloxy)ethanol was varied from 0.2 to 40 mM, using 0.2 mM H_2_O_2_ doses.

Product identification was carried out using an HP 6890 gas chromatographer (Hewlet-Packard, Co., Palo Alto, CA, USA) coupled to a 5973 inert mass selective detector (Agilent Technologies, Inc., Palo Alto, CA, USA) using a fused silica HP-5MS capillary column (30 m × 0.25 mm internal diameter; 0.25 μm film thickness). The oven was set to 70 °C (2 min) and then a linear gradient (5 min) was programmed up to 230 °C, where temperature was kept constant for 3 more min.

### 2.3. Enzyme Kinetics

Catalase activity, determined as oxygen production in the reaction, was measured with a Clark-type electrode calibrated with nitrogen/air and coupled to an oxygraph (Hansatech Instruments Ltd., Pentney, UK). Reactions (500 μL) were started with the addition of H_2_O_2_ to a 50 mM potassium phosphate buffer solution pH 7.0 containing 24–98 nM PaDa-I and ether substrate when indicated. This instrument was previously calibrated with de-oxygenated MiliQ water, to which N_2_ was bubbled for 10 min. Assay solutions were also bubbled with N_2_.

NBD oxidation rate was estimated using a colorimetric assay that follows the production of 4-nitrocatechol (ε_425_ = 9700 M^−1^cm^−1^) [16] in an Agilent 8453 UV-Visible spectrometer (Santa Clara, CA, USA). Reactions (1 mL) were initiated by the addition of 1.0 mM H_2_O_2_ to a 50 mM potassium phosphate buffer solution pH 7.0 containing 5–50 nM PaDa-I, acetonitrile (20% *v*/*v*) and the ether substrate/inhibitor, as indicated.

Kinetic data were fitted to the Michaelis–Menten equation, or the corresponding kinetic model [17,18] when inhibition was present (Equation (1)). Kinetic constants were calculated from a non-linear regression using PRISM 8.2.1 (GraphPad Software, San Diego, CA, USA).

(1) $$v_{O_{2}\;}=\frac{k_{cat\;}\;\left[E_{0}]\;[H_{2}O_{2}\right]}{K_{m}\left(1+\frac{\left[\left(benzyloxy\right)ethanol\right]}{K_{ic}}\right)+\left(1+\frac{\left[\left(benzyloxy\right)ethanol\right]}{K_{iu}}\right)\left[H_{2}O_{2}\right]}$$

Equation (1). Kinetic approach to H_2_O_2_ dismutation inhibition by 2-(benzyloxy)ethanol. *v*_O2_—O_2_ production rate. *k_cat_*—turnover number. [*E_0_*]—total enzyme concentration. *K_m_*—Michaelis–Menten constant. *K_ic_*—competitive inhibition constant. *K_iu_*—uncompetitive inhibition constant.

All experimental determinations in this work are the result of at least three independent experiments carried out at room temperature.

### 2.4. Molecular Docking

The PaDa-I crystallographic structure model (PDB ID: 5OXU) was used to run a 50 ns molecular dynamics simulation as previously described [14] (Ramirez-Ramirez et al., 2020). A total of 243 frames were extracted from the last 20 ns of the simulation, to obtain different conformations of the enzyme for the molecular docking with the substrates. The structures of the substrates were built in GaussView 6.0.16, optimized in Gaussian 16 (Gaussian, Inc., Wallingford, CT, USA) through a quantum mechanics algorithm (level of theory DFT B3LYP/6-311++G**) and prepared in Autodock Tools 1.5.6 enabling bond rotation. Enzyme-substrate flexible docking was performed using Autodock Vina 1.1.2 software [19] and the search was performed through the complete heme-access channel (x = 45.318, y = −33.470, z = −17.149; grid: 12 × 30 × 14). The number of binding modes was set to 10 and graphics results were obtained in PyMol 2.3 (Schrödinger, Inc., New York, NY, USA).

## 3. Results and Discussion

**Scheme 1:**
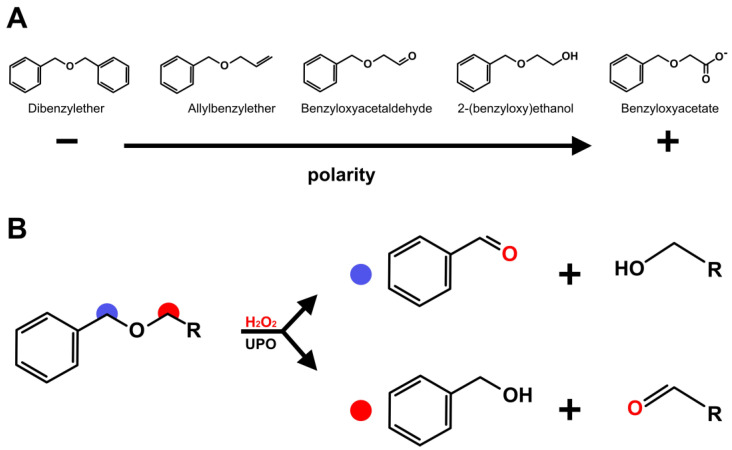
Selected benzyl ethers for this study (**A**), bearing substituents with different polarities. Benzaldehyde or benzyl alcohol is produced after hemiacetal decomposition, depending on which carbon is hydroxylated in the presence of the enzyme (**B**).

In order to study if the chemical nature of substituents in aromatic ethers influenced its oxidation by PaDa-I, a series of five compounds with increasing polarity was selected, as shown in Scheme 1A. Four of the five studied compounds were substrates for the enzyme; no reaction could be detected with benzyloxyacetate. Benzaldehyde and/or benzyl alcohol were identified as products of the enzyme-catalyzed reaction by GC-MS, confirming ether bond cleavage and that the hydroxylation occurred at either of the carbons adjacent to the oxygen atom in the ether functional group (see Scheme 1B).

**Scheme 2:**
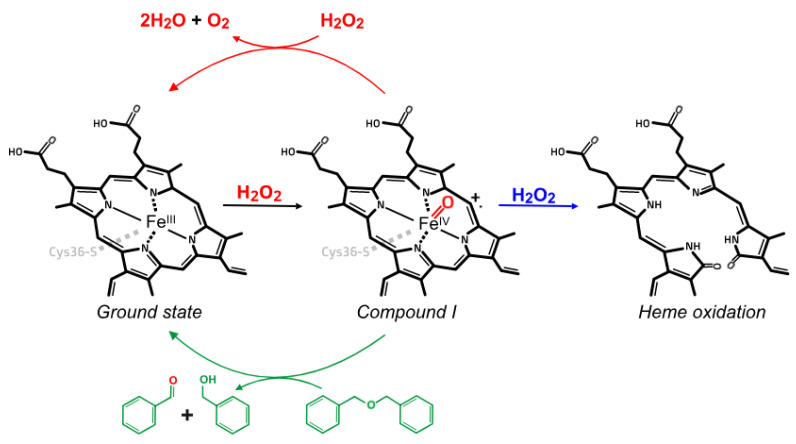
Different reactions occurring during PaDa-I-catalyzed oxidation of ethers. Suicide inactivation (blue) and catalase activity (red) are present during ether oxidation (green).

With the aim to elucidate the effect of different substituents in the aromatic ethers, we attempted to measure kinetic constant kcat and Km. However, these measurements could not be carried out, as the initial reaction rate could not be accurately measured because the reaction halted a few seconds after it started. Although the enzyme concentration was varied, it was not possible to obtain several points in order to accurately measure the initial rate of oxidation. This behavior has been observed by others [9], and we hypothesized it is related to side events occurring in the reaction mixture, mainly the peroxide dismutation and suicide inactivation of PaDa-I (Scheme 2). In order to dissect these events, oxygen production (catalase activity) and residual activity (enzyme inactivation) were monitored, concomitantly with ether oxidation.

Using 2-(benzyloxy)ethanol as a model, we observed that enzyme inactivation occurred along the reaction of ether oxidation. Moreover, both reactions halted and were only reinitiated if H_2_O_2_ was added (Figure 1A,B). Following the assumption that the enzyme was quickly inactivated due to an excess of hydrogen peroxide, we evaluated the effect of dosing the H_2_O_2_ in the reaction, and also the H_2_O_2_ concentration of the doses, on enzyme inactivation and substrate conversion. As expected, as the concentration of H_2_O_2_ doses decreased, the ether consumption increased and enzyme inactivation was slowed. However, eventually the enzyme became completely inactivated. Thus, the efficiency of ether oxidation was measured as the total turnover number (TTN), the mol of substrate converted per mol of enzyme, until the enzyme is completely inactive. TTN for 2-(benzyloxy)ethanol oxidation could be increased by adding H_2_O_2_ in small doses (0.1–0.2 mM) (Figure 1C). The benefits of controlling H_2_O_2_ concentration in terms of increasing the half-life time of the enzyme in the reaction mixture are being studied actively, applying different strategies that combined UPOs with electro-, photo and chemical-catalysis, as well as using enzyme cascade reactions or even fusion enzymes [20].

Moreover, suicide inactivation in other heme-proteins is known to occur, combining one or more molecular events, such as heme degradation, the oxidation of amino acid residues, crosslinking, etc. [21]. Although the information for enzymes belonging to the UPO family is still scarce, it has been reported that for chloroperoxidase from *L. fumago* and UPO the main inactivating event is heme bleaching [22,23].

On the other hand, it is known that some heme-peroxidases, such as the bifunctional enzyme KatG, show catalase activity in the presence of hydrogen peroxide. Consistent with other UPO family members, PaDa-I showed a marginal catalase activity with a turnover ratio of 388 ± 12 s^−1^ and *K_m_* value of 0.757 ± 0.084 mM, which is consistent with the values reported for *wtAae*UPO [23]. In order to quantify the competition between the H_2_O_2_ dismutation and ether oxidation reactions, the apparent kinetic constants for the catalase activity were determined in the presence of 2-(benzyloxy)ethanol, as shown in Table 1.

In the presence of the selected ether, the H_2_O_2_ dismutation rate decreases, reflecting the competition for enzyme intermediates. In fact, upon analysis of the apparent kinetic constants, our data suggest that 2-(benzyloxy)ethanol acts as a linear mixed inhibitor of H_2_O_2_ dismutation, with a *K_ic_* and *K_iu_* of 0.39 and 8.16 mM, respectively (Equation (1)). Thus, it is very likely that catalase activity competes with organic substrate oxidation, particularly if the substrate is not efficiently oxidized by the enzyme (i.e., displays high *K_m_*).

The influence of the substrate concentration was also analyzed. Residual enzyme activity was monitored for all of the studied ethers (Figure 2A). We observed that enzyme inactivation was reduced in the presence of more hydrophobic substrates, which could reflect the affinity of the enzyme for non-polar compounds [14]. TTN was determined for the ethers, and it could be observed that, accordingly with the observations for the inactivation profile, the more hydrophobic substrate (dibenzylether) was the most efficiently converted (Figure 2B).

**Figure 3:**
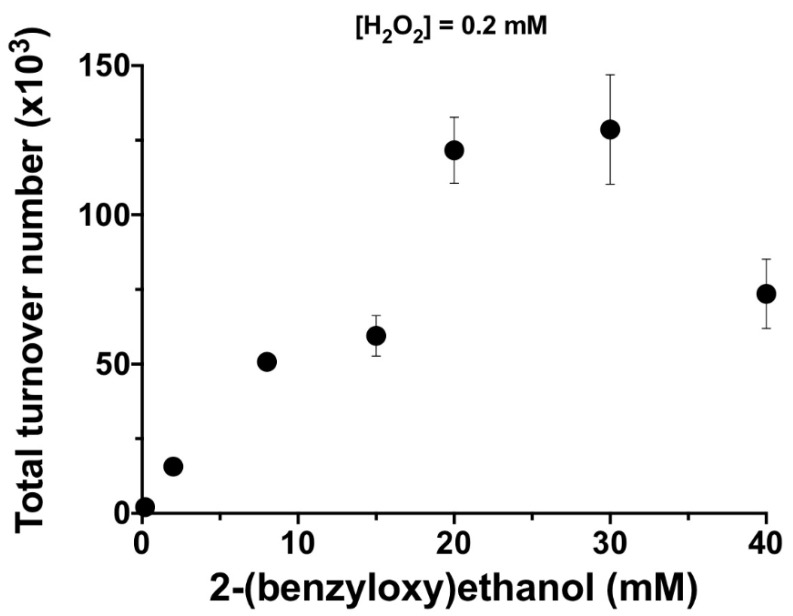
Influence of substrate concentration on total turnover number (TTN), for 2-(benzyloxy)ethanol, determined at various substrate concentrations (0.2–40 mM) and dosing H_2_O_2_ at 0.2 mM.

For 2-(benzyloxy)ethanol and dosing the H_2_O_2_ at 0.2 mM, the TTN was increased from 3000 at 0.2 mM to 129,000 at 30 mM (Figure 3), probably reflecting the low affinity for this substrate and highlighting the importance of increasing substrate concentration for maximizing the catalyst efficiency.

For two of the ether compounds, an unexpected behavior was observed. In the case of allylbenzylether, the enzyme rapidly inactivated after the first H_2_O_2_ addition, leading to low substrate consumption and therefore a low TTN value. Apart from benzaldehyde and benzyl alcohol, MS analysis of the reaction mixture with allylbenzylether revealed the presence of an epoxide, which confirms that olefin oxygenation occurs in addition to ether oxidation [24]. A possible explanation may be related to a previous study with CPO, in which it was shown that terminal olefins form a heme-adduct when carbon atoms in the terminal double bond are oxidized [25]; thus, the heme group became unable to engage in further productive catalytic cycles.

**Figure 4:**
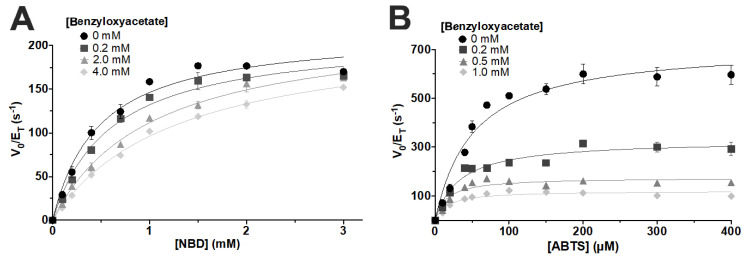
Michaelis–Menten plots for (**A**) NBD peroxygenation and (**B**) ABTS peroxidation in the presence of benzyloxyacetate.

In the case of benzyloxyacetate, no reaction could be detected. In the crystallographic structure of PaDa-I, an acetate molecule binds near the iron atom of the heme group [26]. In order to test if the acetate moiety in the organic ether could be an inhibitor of the enzyme, NBD peroxygenation reactions were performed in the absence and presence of benzyloxyacetate. According to data, benzyloxyacetate behaves as a reversible inhibitor, with *K_i_* = 3.45 mM (Figure 4A). When analyzing the effect of benzyloxyacetate on ABTS peroxidation, an uncompetitive inhibitor behavior was observed, confirming that benzyloxyacetate binds in the heme vicinity, but does not compete with ABTS as the binding site for peroxidative substrates has been proposed to be on the surface of the enzyme [27] (Figure 4B).

**Figure 5:**
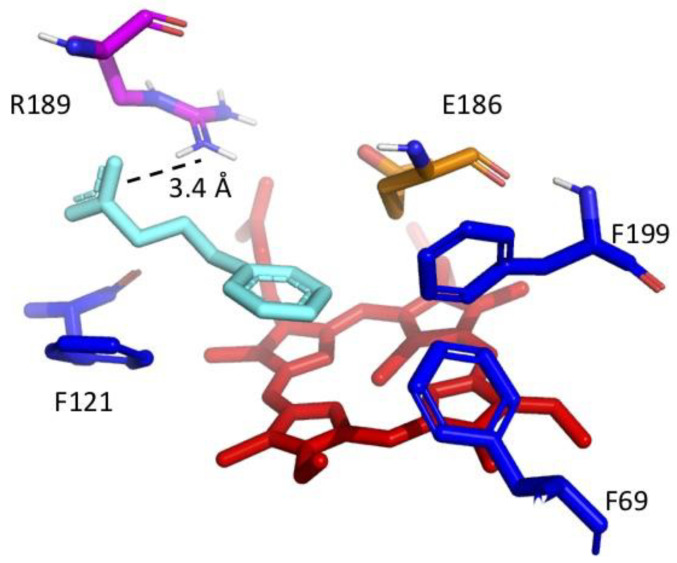
Benzyloxyacetate (cyan) positioned above heme group (red), showing the acetate moiety pointing towards R189 (pink). Surrounding phenylalanine residues (blue) and the catalytic glutamate residue (orange) are also depicted.

Molecular docking studies indicate that 15% of the poses (384 out of 2430) fulfill two conditions: one of the carbons adjacent to the ether oxygen group is at less than 6 Å from the iron heme group; and the score energy is less than −6.5 kcal/mol. In 66% of these 384 poses, it is observed that the aromatic moiety of benzyloxyacetate points towards the heme group, surrounded by the phenylalanine triad (F69, F121 and F199), while the acetate moiety points towards the guanidine group in R189, probably establishing electrostatic interactions (as an example of these poses, see Figure 5). Residue R189 is known to be an auxiliary residue during Compound I formation [26]. However, enzyme inactivation data in the absence and presence of benzyloxyacetate are very similar, thus suggesting that Compound I formation is not hampered in the presence of benzyloxyacetate (Figure 2A). The reason why benzyloxyacetate is not a substrate for the enzyme still needs to be elucidated, as the compound is able to introduce itself in the catalytic cavity (as inferred from its competitive inhibitor behavior) and the aromatic moiety could be oxidized, given that the molecule could approach the activated heme group in Compound I, as suggested by molecular docking.

## 4. Conclusions

Catalytic events and molecular factors during ether oxidation by a recombinant fungal peroxygenase member were studied. Aromatic ethers with more hydrophobic substituents tend to be oxidized more efficiently, strengthening the idea that the *wtAae*UPO and its variant PaDa-I are specialized on the hydroxylation of aromatic compounds. On the other hand, ethers with more polar substituents such as alcohol (in 2-(benzyloxy)ethanol) are less efficiently transformed (1.8-fold less, as measured by TTN). This study demonstrates the feasibility of using a fungal enzyme to degrade such recalcitrant compounds, given that reaction conditions are modified to favor ether oxidation. Regarding secondary, undesirable reactions, we showed that reaction conditions, specifically the dosage of H_2_O_2_ at low concentration, can be controlled in order to enhance the ether transformation efficiency and reduce the rate of side reactions. Synergetic inactivation and low substrate oxidation suggest a suicidal substrate behavior for allylbenzylether, as was previously reported for a related enzyme. The only compound that was not substrate for the enzyme carried the most polar substituent (an acetate group), and according to kinetic and molecular docking, benzyloxyacetate is able to interact with key residues in the active site cavity and behaves as a reversible inhibitor for peroxygenation reactions.

## Figures and Tables

**Table 1:** Apparent kinetic constants estimated for the catalase activity of PaDa-I in the presence of 2-(benzyloxy)ethanol, determined by measuring oxygen production in the reaction.

	2-(benzyloxy)ethanol Concentration (mM)		
	0	2	4
*k_cat_* (s^−1^)	388 ± 12	252 ± 10	231 ± 13
*K_m_* (μM)	757 ± 84	1111 ± 341	2498 ± 331
*k_cat_/K_m_* (M^−1^s^−1^)	5.1 × 10^5^	2.3 × 10^5^	9.2 × 10^4^
